# Endoscopic combined intrarenal surgery in the prone-split leg position for successful single session removal of an encrusted ureteral stent: a case report

**DOI:** 10.1186/s12894-020-00606-5

**Published:** 2020-04-06

**Authors:** Daming Wang, Hongliang Sun, Lei Chen, Zhiqi Liu, Dazhao Zhang, Dexin Yu, Demao Ding

**Affiliations:** Department of Urology, The Second Affiliated Hospital of Anhui Medical University, Hefei, 230601 China

**Keywords:** Prone-split leg position, Encrusted ureteral stent, Endoscopic combined intrarenal surgery

## Abstract

**Background:**

The most serious complication of ureter stent is long-term retention of ureteral stent and stone formation around the stent.

**Case presentation:**

A 51-year old female patient with left ureteral stent placed 2 years before developed both pyelic and vesical stones on the two ends of the double J was admitted to our hospital. Intravesical lithotripsy, retrograde ureteroscopy, and percutaneous nephrolithotripsy were performed with the patient in the prone split-leg position. All the stones and the ureteral stent were successfully removed in a single session.

**Conclusions:**

Combined endoscopic techniques in the prone split-leg position can effectively and safely manage severely encrusted stents.

## Background

Ureteral stent was introduced in 1974 to relieve ureteral obstruction and play an important role in the advance of endourology [1]. Nowadays, ureteral stent was widely used in ureteral calculi, stricture operation, ureteral injury and relieving tumor compression. However, the potential complications of ureteral stent placement include hematuria, infection, pain, ureteral injury, displacement, stone formation and encrustation. The ureteral stent should be implanted by fully trained personnel to avoid improper ureteral stent operation. It is suggested that cystoscopy and / or X-ray should be used to check the ureteral stent for signs of encrusted and normal function. Ureteral stent is not a permanent retention device and should not be placed in the body beyond the specified time. The delayed removal of ureteral stent increases the incidence of encrusted ureteral stent with prolonged retention time. Bostanci et al. reported that the incidence of the patients who forgot to take out the stent with encrustation was about 0.64% [2]. The initial recommendations for the management of an encrusted stent include shock wave lithotripsy, ureteroscopy lithotripsy (URSL) and percutaneous nephrolithotripsy (PCNL). However, if the stone around the stent tube is too large to be completely removed, it may need to be treated by extracorporeal lithotripsy or surgery many times, which can cause great pain and financial burden to the patients. This report presents an interesting case of successful removal of an encrusted ureteral stent in a single session by endoscopic combined intrarenal surgery (ECIRS).

## Case presentation

**Fig. 1:**
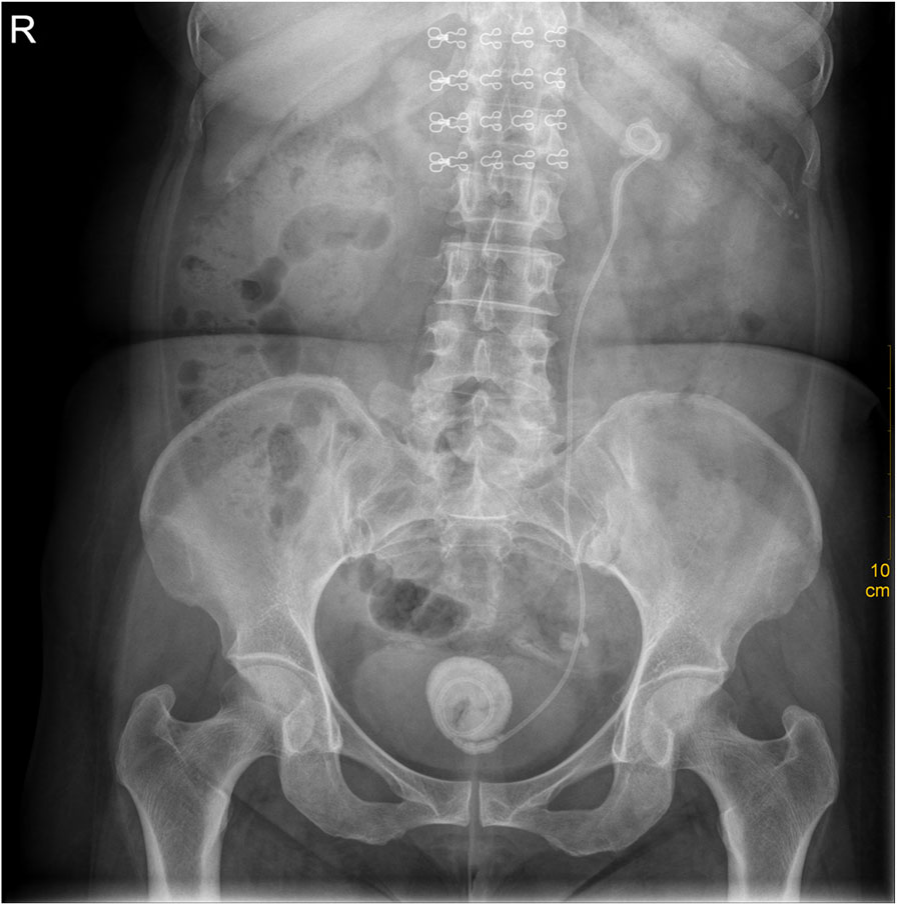
The abdominal radiograph showed that circular encrustations completely encasing both of the pigtail portions of the indwelling ureteral stent

**Fig. 2:**
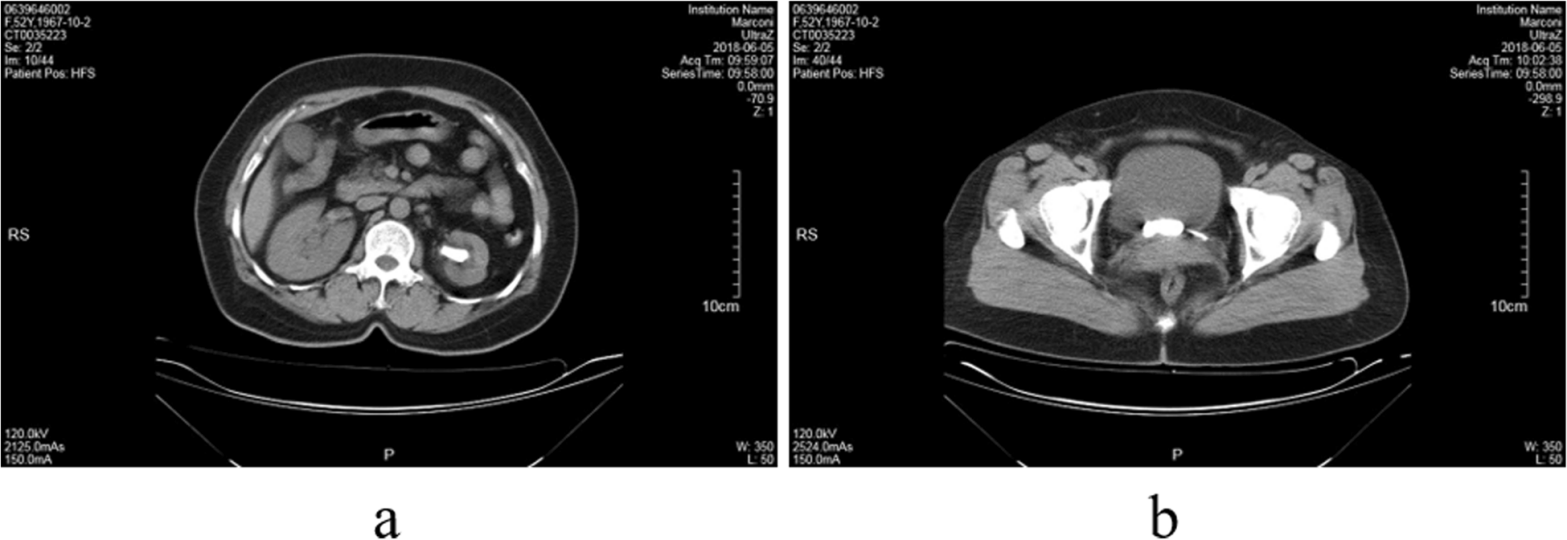
Figure 2**a**: Single stone in the renal pelvis, wrapped around the upper part of the stent. Figure 2**b**: Large oval stone in bladder, wrapped around the end of stent tube

A 51-year-old female patient underwent left URSL 2 years ago. However, she lose follow-up before complete removal ureteral stent. Recently, she presented to the department of urology with left lumbar pain. She suffered from urgency and frequency of urination and without the history of other diseases and operation. Physical examination: Body mass index was 25.12 kg/m^2^, left renal percussive pain was positive. An abdominal radiograph showed that both end of ureter stent encrusted with the stone (Fig. 1). Computed tomography (CT) examination reported that a single large stone, located in the renal pelvis and bladder, wrapped around the end of the stent tube, with a length of more than 2 cm (Fig. 2). The CT Hounsfield Unit value of left intrarenal calculi was 645HU, and that of bladder calculi was 1150HU. Urinalysis testing revealed a red blood cell count of > 100/hp. and a white blood cell count of 120/hp. Urine bacterial culture suggested the presence of *Escherichia coli*. Other routine laboratory examinations were all within normal limits.

**Fig. 3:**
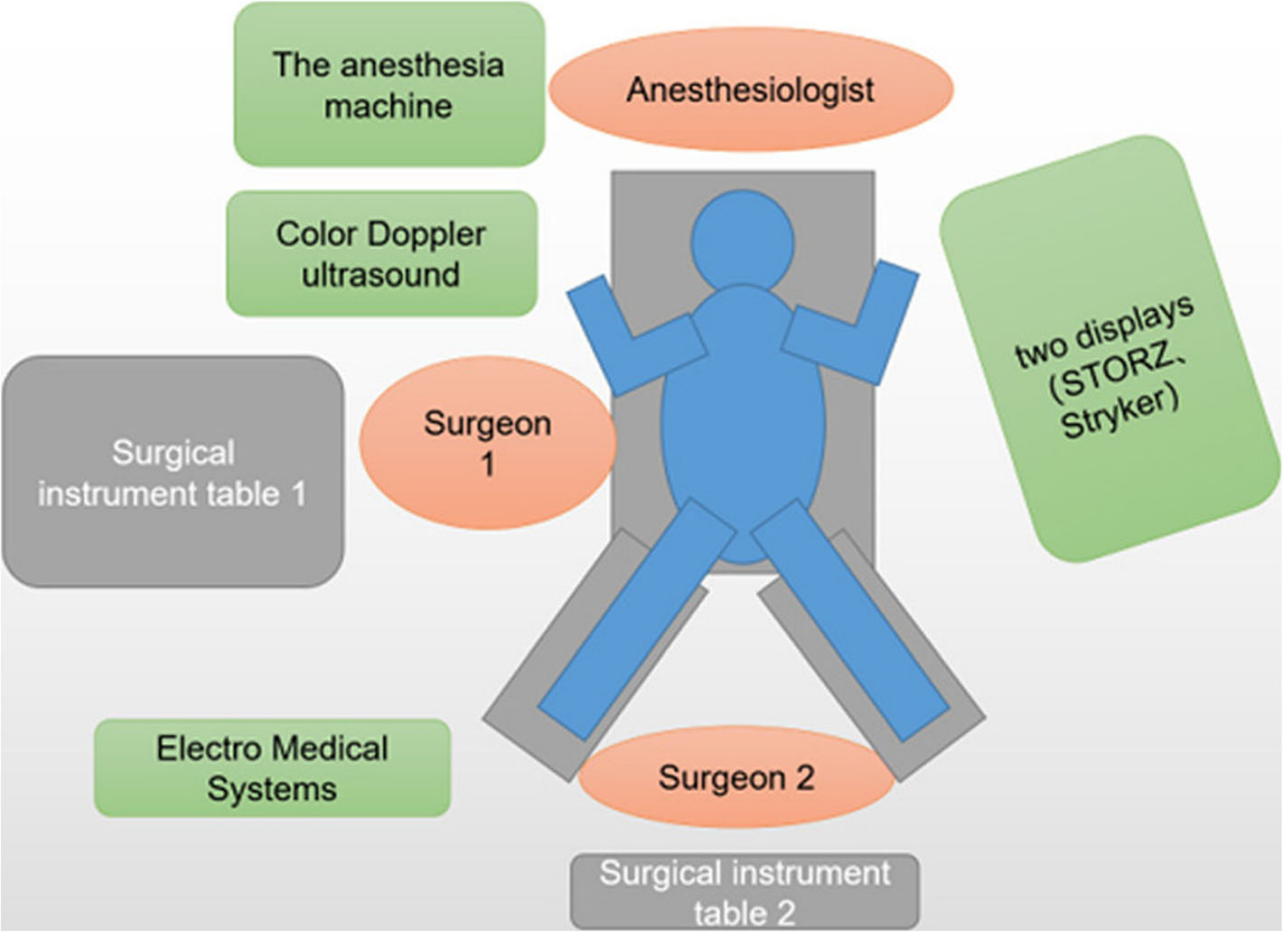
The schematic diagram shows the position of the patient in prone-split leg position, the position of the surgeon and the placement of the instruments

**Fig. 4:**
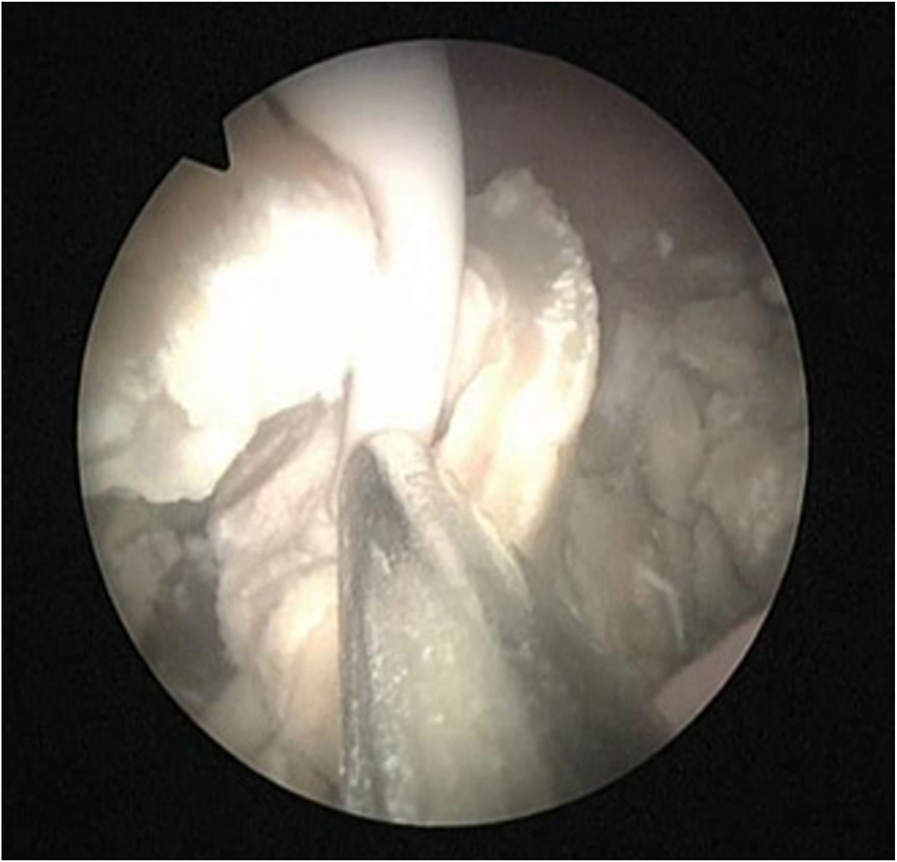
The distal end of the ureteral stent was severe encrusted, the diameter is about 2 cm. Pneumatic ballistic gravel through nephroscopes into the bladder

**Fig. 5:**
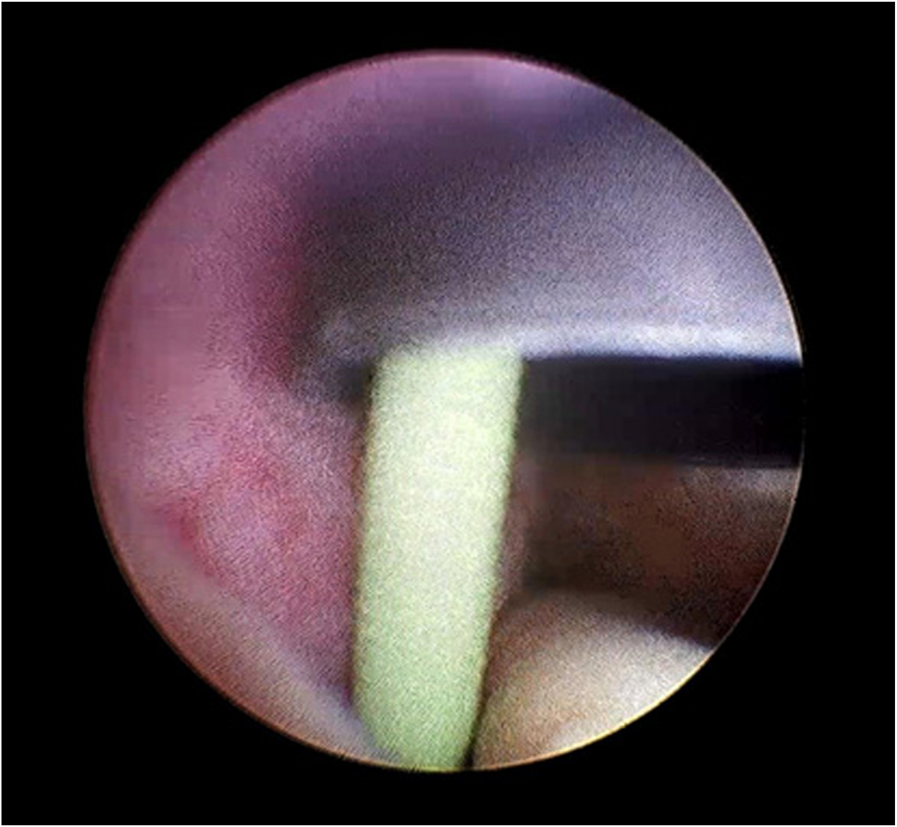
The ureteroscope enters the left renal pelvis along the stent tube and guide wire, ureteroscope to monitor the dilation and establishment of puncture channel in renal pelvis

**Fig. 6:**
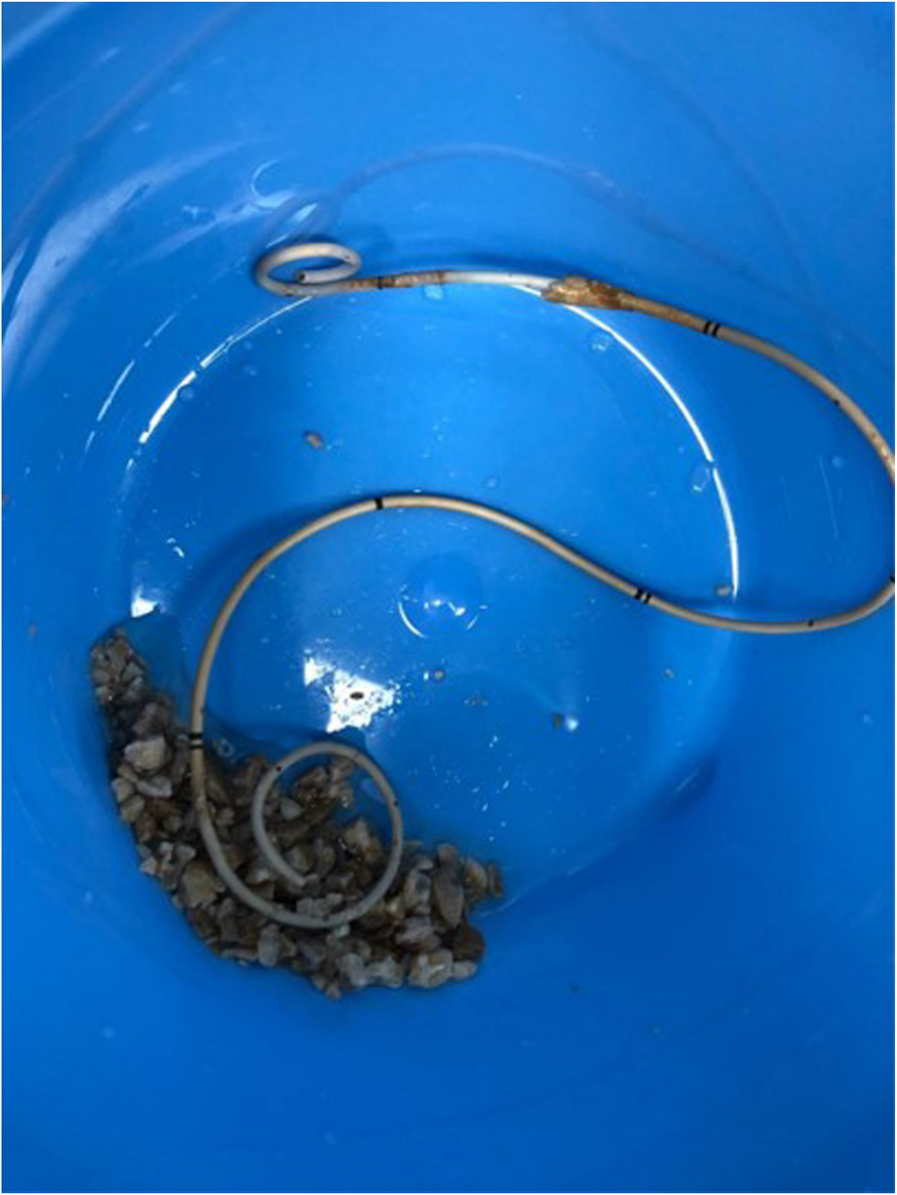
Ureteral stent and stone were removed completely


According to the results of urinary bacterial culture, the third generation cephalosporins was selected for anti-infection treatment for 5 days. Stent removal was attempted by ECIRS under general anesthesia. The patient was oriented in the prone-split leg position throughout the operation, and then we performed retrograde operation and PCNL (Fig. 3). The operation was performed by two urologists at the same time, one performed PCNL, the other performed transurethral retrograde surgery. First, Surgeon 2 was performed nephroscope (f20, Wolf, Germany) combined with resectoscopes sheath (f26.5) to enter bladder, observed the stent encrustation and located the ureteral orifice. The distal end of the ureteral stent was highly encrusted, the diameter was about 2 cm. Pneumatic ballistic gravel (Electro Medical Systems, Swiss LithoClast® Maste, Nyon, Switzerland) entered into the bladder through nephroscopes (Fig. 4), and the stone fragment was rushed out. The Surgeon 2 retrograde entered into the left ureteral orifice with a ureterscope (Karl Storz, Tuttlingen, Germany), and then entered the left renal pelvis along the stent tube and guide wire. The ureter was in good condition without stricture and damage, and there were no encrusted in the middle of stent tube. Under ultrasound guidance, surgeon 1 chose the left posterior axillary line under 12 ribs as the puncture position and established the nephroscope channel (f22). Meanwhile, surgeon 2 monitored the dilation and establishment of puncture channel with ureterscope to avoid kidney injury caused by too deep puncture channel (Fig. 5). Surgeon 1 entered the renal pelvis with a nephroscope (f20, Wolf, Germany). Ultrasonic lithotripter (ultrasonic energy 80%, duty cycle 70%, Electro Medical Systems, Swiss LithoClast® Maste, Nyon, Switzerland) removed the stent encrustation in the renal pelvis, broke the stones into smaller fragments. The fragments were drawn from the channel with negative pressure (Additional file 1). After removal of intrarenal calculi, the stent tube was removed completely by foreign body forceps through the channel. No residual stone was found in the renal pelvis and ureter (Fig. 6). Finally, a new stent tube (f6, 28 cm, urovision) was placed into the ureter, and ECIRS was used to ensure the correct position of the stent tube. The total operation time was 74 min. There was no complication after the operation. No residual stone was found in postoperative reexamination imaging. The nephrostomy tube was removed five days after surgery. The ureteral stent was removed one month later. An analysis of the encrusting material indicated that it was a calcium phosphate calculus.

**Additional file 1.** A video of endoscopic combined intrarenal surgery: First, surgeon perform pneumatic ballistic lithotripsy in bladder. Second, ureteroscopy is used to examine the ureter to the renal pelvis and to monitor the establishment of percutaneous nephrolithotomy channel. Finally, ultrasonic lithotripter remove the stent encrustation in the renal pelvis. Foreign body forceps is used to remove the stent tube.


## Discussion and conclusion

With the improvement of medical technology and the application of new biomaterials and ureteral stent coatings, the incidence of infection and encrustation has declined significantly [3]. However, many studies indicated that ureter stent indwelling time is highly associated with the incidence of encrustation [4]. El-Faqih et al. reported that the stent encrustation rate increases from 9.2% for an indwelling time of less than 6 weeks to 76.3% for more than 12 weeks [5]. Severe encrustation with stone formation can lead to urinary tract obstruction, infection, and kidney damage. Hence, stent encrustation must choose a reasonable surgical method to avoid ureteral injury, stent rupture and multiple operations. The case we reported may provide an effective surgical approach to remove an encrusted ureteral stent.

Acosta-Miranda et al. reported that according to the imaging examination, the stent encrustation is graded to facilitate the selection of treatment methods [6]. This classification is based on the stone size, location, and degree of stent encrustation. According to the above grading method, the cases reported in this study are grade IV. The diameter of circular encrustations at both ends is greater than 2 cm. Many studies have pointed out that different methods of lithotripsy should be used at the upper and lower ends of this case [6, 7]. However, how to remove the stent tube and stone by a combination of different lithotripsy methods in one position.

If the patient want to perform operation, the first step is to perform cystoscopic lithotripsy and URSL in the lithotomy position, and then to perform PCNL in the prone position. It may cause prolonged operation time, repeated placement of body position during operation, fracture of stent tube and residual stone. In the prone-split position, ECIRS has obvious advantages in the treatment of severe stent encrustation. Avoiding the repeated placement of body position during an operation to ensure the continuity of operation. The combination of two kinds of endoscopes in the same operation has good complementarity. For renal pelvis stone, the combination of upper and lower endoscopy increases the operation field of vision and expands the operation angle. As a result, the ECIRS increases the extent of the rubble. Besides, in the prone position, the ureteral channel tends to be straight under the action of gravity, making it easier for ureteroscope to enter the upper ureter and renal pelvis [8]. Under monitor of the ureteroscope, the dilation of the percutaneous nephroscope puncture channel can avoid renal injury [9]. The mutual drainage channel can reduce the pressure in the renal pelvis and reduce the extravasation of the perfusion during the high-pressure washing of the lithotripsy. Thereby reduceing the risk of postoperative infection. Different from other reports [10–12], this case is treated in one position combined with intravesical lithotripsy, retrograde ureteroscopy and percutaneous nephrolithotripsy. The whole process of puncture and removal of stent tube is monitored. In addition, the operation is successful once, avoiding reoperation, repeatedly placing the body position, stent rupture and open bladder operation. Tetsuya Isero et al. reported that a case of severe stent encrustation is successfully removed by flexible ureteroscopy and miniature nephroscopy in the prone split-leg position [13]. Compared with the Galdakao-modified supine Valdivia position [10, 14], the prone split-leg position has the following advantages: (1) The operator is familiar with the position and easy to place. The percutaneous nephroscopy puncture space is larger and the risk of visceral injury is smaller. (2) There are no obesity and cardiopulmonary disease in the patients. But the prone position had no significant effect on their cardiopulmonary function. (3) Avoiding long-term elevation of lower limbs and compression of blood vessels. Most of the stent encrustation are phosphate (struvite) and calcium phosphate crystals [15]. The analysis of this case is calcium phosphate calculus. Continue to use sensitive antibiotics and reduce urine pH value after operation. For patients with stent placement, strict follow-up can effectively prevent stent from being forgotten and stone formation.

In summary, a case of severe ureteral stent encrustation is successfully treated by endoscopy combined in one position. The stone and stent are removed completely in one operation, avoiding the change of body position and reoperation. The removal of stent is completely monitored to avoid rupture of stent and ureteral injury. The combination of upper and lower endoscopy can enlarge the operative field of vision and reduce the injury. Thus, ECIRS in the prone-split leg position could achieve successful and safe management of severe encrusted stents.

## Data Availability

The datasets used during this study available from the corresponding author on reasonable request.

## Abbreviations

URSL: Ureteroscopy lithotripsy
PCNL: Percutaneous nephrolithotripsy
FURSL: Flexible ureteroscopy lithotripsy
ECIRS: Endoscopic combined intrarenal surgery
CT: Computed tomography
